# The Science of Scale for Violence Prevention: A New Agenda for Family Strengthening in Low- and Middle-Income Countries

**DOI:** 10.3389/fpubh.2021.581440

**Published:** 2021-03-19

**Authors:** Yulia Shenderovich, Jamie M. Lachman, Catherine L. Ward, Inge Wessels, Frances Gardner, Mark Tomlinson, Daniel Oliver, Roselinde Janowski, Mackenzie Martin, Kufre Okop, Hlengiwe Sacolo-Gwebu, Lindokuhle L. Ngcobo, Zuyi Fang, Liane Alampay, Adriana Baban, Ana A. Baumann, Regina Benevides de Barros, Samuel Bojo, Alexander Butchart, Wilmi Dippenaar, Amon Exavery, Xiangming Fang, Ida Ferdinandi, Heather M. Foran, Nina Heinrichs, Judy Hutchings, Daisy Kisyombe, Greta Massetti, Jaromir Mazak, Henry Mbuyi, Pratibha Singh, Kenneth Polsky, Sabine Rakotomalala, Marija Raleva, Richard Savo, Lucie Cluver

**Affiliations:** ^1^Department of Social Policy and Intervention, University of Oxford, Oxford, United Kingdom; ^2^Centre for the Development and Evaluation of Complex Interventions for Public Health Improvement (DECIPHer), School of Social Sciences, Cardiff University, Cardiff, United Kingdom; ^3^MRC/CSO Social and Public Health Sciences Unit, University of Glasgow, Glasgow, United Kingdom; ^4^Department of Psychology, University of Cape Town, Cape Town, South Africa; ^5^Department of Global Health, Institute for Life Course Health Research, Stellenbosch University, Stellenbosch, South Africa; ^6^School of Nursing and Midwifery, Queens University, Belfast, United Kingdom; ^7^Catholic Relief Services, Baltimore, MD, United States; ^8^Clowns Without Borders South Africa, Cape Town, South Africa; ^9^Department of Psychology, Ateneo de Manila University, Quezon City, Philippines; ^10^Department of Psychology, Babes-Bolyai University, Cluj-Napoca, Romania; ^11^Brown School of Social Work, Washington University in St. Louis, St. Louis, MO, United States; ^12^Division of Global HIV and TB, Centers for Disease Control and Prevention, Atlanta, GA, United States; ^13^Agency for Research and Development Initiative, Juba, South Sudan; ^14^Department of Injury and Violence Prevention, World Health Organization, Geneva, Switzerland; ^15^The Seven Passes Initiative, Wilderness, South Africa; ^16^Pact Tanzania, Dar es Salaam, Tanzania; ^17^School of Public Health, Georgia State University, Atlanta, GA, United States; ^18^UNICEF Montenegro, Podgorica, Montenegro; ^19^Institute for Psychology, Universitat Klagenfurt, Klagenfurt, Austria; ^20^Department of Psychology, University of Bremen, Bremen, Germany; ^21^School of Psychology, Bangor University, Bangor, United Kingdom; ^22^Pact Eswatini, Mbabane, Eswatini; ^23^Division of Violence Prevention, Centers for Disease Control and Prevention, Atlanta, GA, United States; ^24^Schola Empirica, Prague, Czechia; ^25^Faculty of Arts, Charles University, Prague, Czechia; ^26^Catholic Relief Services DRC, Gombe, Democratic Republic of Congo; ^27^Emmanuel Hospital Association, New Delhi, India; ^28^Global Partnership to End Violence Against Children, World Health Organization, Geneva, Switzerland; ^29^Department of Child and Adolescent Psychiatry, St. Cyril and Methodius University Skopje, Skopje, Macedonia; ^30^Catholic Relief Services Zimbabwe, Harare, Zimbabwe; ^31^Department of Psychiatry and Mental Health, University of Cape Town, Cape Town, South Africa

**Keywords:** violence—prevention and control, violence against children and adolescents, parenting, implementation science, parenting (MeSH)

## Abstract

Ending all violence against children by 2030 is a core part of Sustainable Development Goals 5 and 16. A number of promising violence reduction strategies have been identified in research studies. However, we lack an understanding of the implementation and impact of these programs in respect to their delivery at a large scale or within existing service systems, particularly in low- and middle-income countries (LMICs). We advocate for greater collaboration between researchers, policymakers, donors, governments, non-governmental organizations, and program managers and staff to study how violence prevention programs operate on a large scale. We describe a new initiative aiming to foster such collaborations in the field of family strengthening programs.

## Introduction

Over a billion children experience violence each year, with a disproportionate number of those in the Global South (1). Violence against children has been linked to a multitude of immediate and long-term negative health outcomes and substantial economic costs. It violates the UN Convention on the Rights of the Child, and ending “abuse, exploitation, trafficking and all forms of violence against and torture of children” and “all forms of violence against all women and girls in the public and private spheres” are specific targets of the 2030 Sustainable Development Goals (SDG 16.2 and SDG 5.2).

There are a number of evidence-informed and promising interventions for preventing and reducing violence against children, such as those detailed in the World Health Organization-led INSPIRE framework (2–8). To achieve the SDGs, these interventions need to be scaled up and evaluated at the population level (9). However, the current evidence base on scaling up violence prevention strategies and evaluating their scale-up is limited, particularly in LMICs [see (10) on scaling social norm change interventions and (11) on scaling early childhood development].

There is a growing evidence base of research on parenting programs, including promising results in diverse LMIC settings [e.g., (12)] and studies on scaling parenting programs in high-income countries [e.g., (13)], but studies examining scaling parenting programs in LMICs are urgently needed to inform practice in the field.

While randomized trials are important for understanding intervention effectiveness, they are often conducted under circumstances that do not fully reflect real-world delivery. For instance, the external validity of study results is limited when particularly competent and well-resourced organizations take part in research studies. Thus, initially effective interventions may face a high risk of failure when taken to scale (14).

## Challenges of Scale-Up

Successful scale-up encompasses expanding program coverage and quality to larger populations or areas and embedding program delivery into lasting systems (15). The successful scale-up of violence prevention programs is challenging for a variety of reasons. As violence against children is an issue that cuts across sectors such as health, social welfare, education, and justice, violence prevention programs often require multi-sectoral cooperation to fit within existing service delivery systems that face substantial resource constraints. In addition to the financial commitment and material resources necessary to sustain programs, implementing agencies also need the structures and technical capacity to deliver interventions with fidelity and quality. Although task-shifting through community or lay workers is often seen as a solution for rapid scale-up of low-cost service provision in LMICs, the training, supervision, and retention of such workers can be costly and requires effective organizational management (16). Moreover, frontline service providers are frequently overburdened and underpaid, resulting in high turnover or poor service delivery in low-resource settings where the need is greatest.

We also have to accept that program implementers often resort to *ad hoc* and reactive adaptation—for example, to simplify or reduce costs of social interventions—thus deviating from the evidence established in randomized trials. Interventions may either mature and evolve to suit the cultural and organizational context better, or they may drift and include counterproductive changes. Substantial research has been conducted on cultural adaptation, and scholars have identified the importance of adapting interventions for implementation in routine settings. However, beyond the formal studies of adaptation, the vast majority of adaptations are *ad hoc* and not reported (17). There is also scarce evidence on the impact of these informal adaptations made during scaling up, highlighting a need to continue researching interventions as they are delivered more widely and within new systems. Furthermore, measuring violence is challenging, for instance due to stigma and related under-reporting (18, 19), and reliable administrative data on the implementation and outcomes of services related to violence against children are rarely available. Thus, continuously improving and conducting research on violence prevention programs at a large scale or within scaled-up programs presents formidable challenges.

## Scale-Up of Parenting Evaluation Research Study

A new collaboration between researchers and implementing agencies is the Parenting for Lifelong Health Scale-Up of Parenting Evaluation Research (SUPER) study (20). This cross-sectoral collaboration is working to harness implementation science in order to study and maximize the scale-up and effectiveness of parenting interventions that reduce violence against children and improve child well-being. It focuses on the recent rapid dissemination of two Parenting for Lifelong Health (PLH) parenting programs throughout LMICs, PLH for Young Children (2–9 years), and PLH for Parents and Teens (10–17 years) (21). PLH is a suite of evidence-based parent training programs based on social learning theory and designed to reduce violence against children. Originally developed based on evidence of key parenting program components (3, 22), and tested in South Africa, PLH programs have demonstrated positive impacts in randomized trials in reducing violence against children, improving child development and mental health, and increasing family economic well-being (23–26).

The PLH programs involve a series of weekly group meetings and/or home visits based on the structure provided by the intervention manuals. Since their initial testing, PLH for Young Children and PLH for Parents and Teens have been rapidly disseminated to over 25 LMICs at varying degrees of scale, with additional trials forthcoming in other countries. A range of government, non-governmental, and parastatal agencies deliver PLH, often within large-scale donor-driven initiatives in combination with other social services and implementation packages. For example, in several countries in Sub-Saharan Africa, PLH has been delivered within the DREAMS projects funded by PEPFAR-USAID focusing on adolescent girls and young women. In some settings, PLH programs have been integrated into existing government packages of services, such as the Philippines' government conditional cash transfer system and the Thailand public health promotion system (27–29). By the end of 2022, PLH programs are expected to reach approximately half a million families in Sub-Saharan Africa, Eastern Europe, South and Southeast Asia, and the Caribbean. In response to the COVID pandemic, in 2020 a set of parenting tips based on the PLH programs has been made freely available and accessed by estimated 136.1 million people through a global collaboration that includes international agencies, as well as governments and NGOs (30).

**Figure 1:**
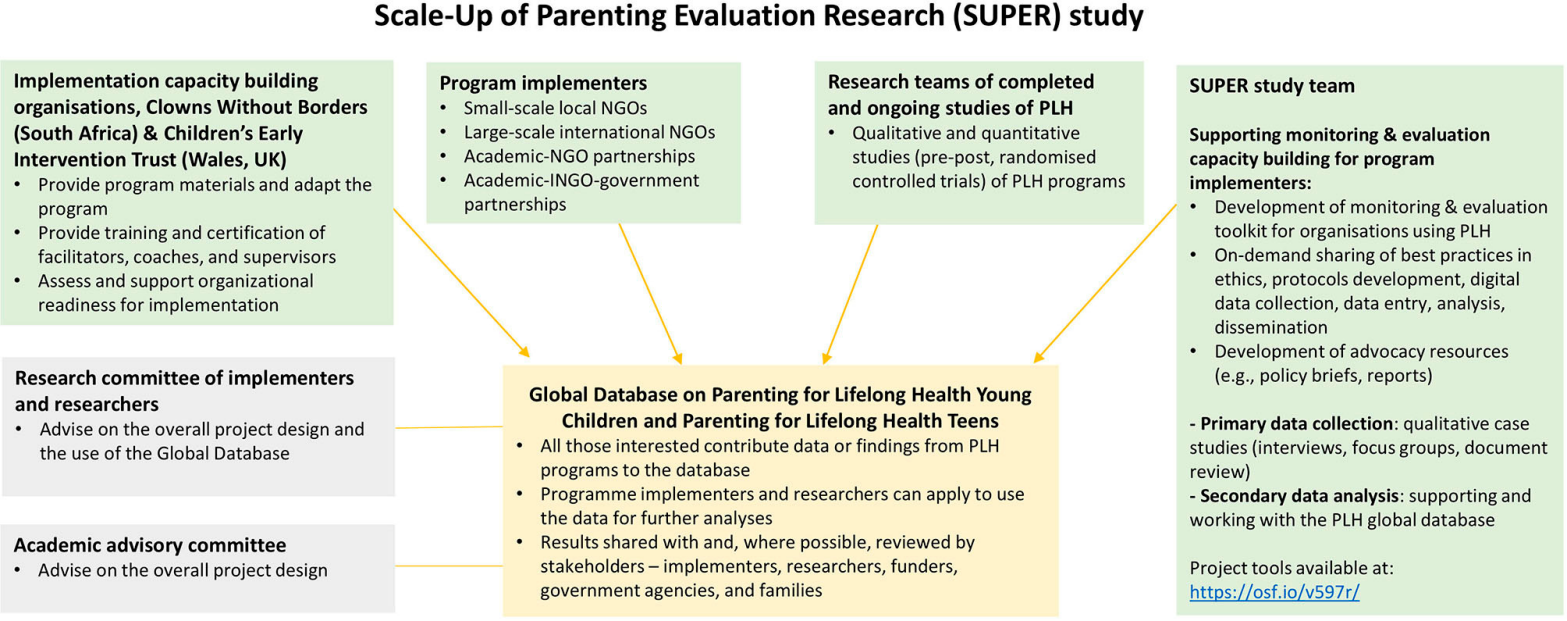
Overview of the collaborations linked to the SUPER study.

The SUPER study represents an opportunity for researchers, policymakers, donors, implementing agencies, and community stakeholders from within and outside LMICs to collaborate in order to deepen our understanding of the scale-up of family-based interventions in low-resource settings (see Figure 1 for an overview). The study aims to examine processes for effectively adopting, implementing, adapting, monitoring, and disseminating PLH programs. Translational research is a particular focus of the SUPER study, investigating processes and mechanisms for successful delivery of evidence-informed interventions through existing service delivery structures. It also examines program transferability across cultures and contexts, and how variations in design, implementation approach, and participant engagement are associated with family and program-level outcomes. Finally, it addresses questions around financial and human resources needed to sustain scale-up and identifies overall trends and factors related to successful scale-up. Analyses of program costs may also be used to assess and improve the performance of PLH programs to maximize child well-being.

The SUPER study will use innovative research methods, drawing on ongoing service delivery data, as well as qualitative and quantitative research data collection that includes trial data, to generate quality evidence that will inform programming. The study will include data at the individual, familial, organizational, and national levels. It will focus on insights for action, and—being mindful of the burden on service providers—aim to uphold the principle to “only collect data you can commit to use” [(31), p. 26]. Another major component of the study is mutual learning and ongoing program improvement, through which researchers gain insights about implementation at scale while implementing agencies gain skills in robust monitoring strategies and evidence generation. Collaborators will also work together to maximize the dissemination and utilization of research at community, national, and international levels. The SUPER study is thus offering a window of opportunity to close the gap between research and practice in the area of violence prevention and enhancement of child well-being through parenting support.

## Discussion

Achieving SDG targets 5.2 and 16.2 by ending violence against children will require researchers, technical experts, policymakers, governments, donors, implementing agencies, local program managers, and community stakeholders to work closely together to answer challenging questions regarding how programs can both be effective and sustained at scale. Through these collaborations, we may begin to learn how we can best prevent violence against children and support frontline violence prevention. As a global community, it is essential that we advance scientific knowledge on what works, for whom, and how, to improve the well-being of children and their families at scale; the PLH SUPER study aims to act on this agenda.

## Data Availability Statement

The original contributions presented in the study are included in the article/supplementary material, further inquiries can be directed to the corresponding author. Study materials are available on the project Open Science Framework page: https://osf.io/v597r/.

## Author Contributions

YS, LC, and JL have led the writing of the paper. All authors have contributed to conceptualizing the paper, reviewed the manuscript, approved the final manuscript as submitted, and agree to be accountable for all aspects of the work.

## Conflict of Interest

LC, CW, JL, JH, and FG were involved in the development of the PLH programs. YS, JL, and IW worked on the PLH trials in South Africa and based their doctoral work on these. RJ and MM's current master's work is based on PLH. The work of LC, YS, IW, CW, JL, FG, and SB is partly funded by the UKRI GCRF Accelerating Achievement for Africa's Adolescents Hub. The work of JL and MM has been supported by a GCRF Center Hub Grant. The work of LC, YS, IW, CW, JL, FG, HF, ABab, MR, NH, and RJ has been partly funded by grants under the European Research Council's Horizon 2020 program. The work of JL, CW, and FG has also been funded by UNICEF Thailand, and the work of JH, LA, JL, CW, and FG by UNICEF Philippines. Further, JL is the former Executive Director and receives income as a current Senior Advisor and PLH Trainer at Clowns Without Borders South Africa (a non-profit organization responsible for PLH implementation). LN is Co-Director of Clowns Without Borders South Africa. CW reports grants from the National Research Foundation of South Africa and the World Childhood Foundation. RJ reports a grant from the University of Cape Town during the conduct of the study. Outside the submitted work, SB reports income from IntraHealth Inc. ABut is the co-chair of the INSPIRE: Seven strategies for ending violence against children technical package implementation working group and is a lead author of the package. NH reports involvement in two other parenting programs—Triple P and Parent-Child Interaction Therapy. JH receives occasional income as a PLH Trainer. Outside the submitted work, JM's organization, Schola Empirica, receives grants from the European Social Fund. Further, JM receives income from these grants in his role as program evaluator. The remaining authors declare that the research was conducted in the absence of any commercial or financial relationships that could be construed as a potential conflict of interest.
